# Reactivity of disulfide bonds is markedly affected by structure and environment: implications for protein modification and stability

**DOI:** 10.1038/srep38572

**Published:** 2016-12-12

**Authors:** Maryam Karimi, Marta T. Ignasiak, Bun Chan, Anna K. Croft, Leo Radom, Carl H. Schiesser, David I. Pattison, Michael J. Davies

**Affiliations:** 1 The Heart Research Institute, 7 Eliza St, Newtown, NSW, 2042, Australia; 2 Faculty of Medicine, University of Sydney, NSW, 2006, Australia; 3 Department of Biomedical Science, Panum Institute, University of Copenhagen, Blegdamsvej 3, Copenhagen 2200, Denmark; 4 School of Chemistry, University of Sydney, Sydney, NSW 2006, Australia; 5 Department of Chemical and Environmental Engineering, University of Nottingham, Nottingham NG7 2RD, Great Britain; 6 School of Chemistry, Bio21 Molecular Science and Biotechnology Institute, The University of Melbourne, Victoria 3010, Australia

## Abstract

Disulfide bonds play a key role in stabilizing protein structures, with disruption strongly associated with loss of protein function and activity. Previous data have suggested that disulfides show only modest reactivity with oxidants. In the current study, we report kinetic data indicating that selected disulfides react extremely rapidly, with a variation of 10^4^ in rate constants. Five-membered ring disulfides are particularly reactive compared with acyclic (linear) disulfides or six-membered rings. Particular disulfides in proteins also show enhanced reactivity. This variation occurs with multiple oxidants and is shown to arise from favorable electrostatic stabilization of the incipient positive charge on the sulfur reaction center by remote groups, or by the neighboring sulfur for conformations in which the orbitals are suitably aligned. Controlling these factors should allow the design of efficient scavengers and high-stability proteins. These data are consistent with selective oxidative damage to particular disulfides, including those in some proteins.

Disulfide bonds play a critical stabilizing role in many protein structures by forming cross-links between different regions of polypeptide chains. 21% of the ~90,000 protein structures of the Protein Data Bank contain at least one disulfide bridge, and this incidence is considerably higher in many structural and extracellular matrix proteins and receptors. Furthermore, cysteines involved in disulfide bonds are the most evolutionarily-conserved amino acids (unlike free cysteine), and in 99% of cases these are only replaced as pairs^1^. Disulfides serve as (largely permanent) molecular “staples” that direct and stabilize the three-dimensional structure of proteins, and determine the distance and angle constraints between the joined cysteine residues, therefore stabilizing the folded state with respect to the unfolded form^2^. Disulfides can also play a functional and transient role in enzyme activity^3^,^4^, such as in redox-active proteins that undergo thiol-disulfide interconversion (e.g., the thioredoxin superfamily). In these cases, the disulfide formed on oxidation of vicinal thiols, is often found in (low abundance) high-energy strained conformations (e.g., the so-called +/− RH Hook and –RH Staple configurations), which are believed to facilitate rapid reduction back to the di-thiol form^5^,^6^. Such redox-active disulfides have been shown to be important for normal cellular function, with perturbations postulated to be involved in pathological conditions that are characterized by abnormal/altered redox states, including ageing, cardiovascular disease, some cancers, asthma, rheumatoid arthritis, cystic fibrosis and multiple neurodegenerative diseases (e.g., Alzheimer’s, Parkinson’s, Huntington’s, Creutzfeldt-Jakob disease)^7^.

Chronic inflammation is a well-established contributor to tissue damage associated with disease^8^. Chronic inflammation can arise from excessive or continued stimulation of immune cells from ongoing infection and/or incomplete removal of stimulants (e.g., particulate matter, oxidized materials)^9^, with this resulting in ongoing damage to tissues, with subsequent poor repair and remodelling (reviewed)^8^. Stimulation of neutrophils, monocytes and macrophages results in the assembly and activation of complexes that generate oxidants, including NADPH oxidases (NOXs) that generates superoxide radicals (O_2_^**−**·^) and hence H_2_O_2_ by dismutation, and nitric oxide synthases that produce nitric oxide (NO^·^)^10^,^11^. Reaction of O_2_^**−**·^ with NO^·^ generates the powerful oxidant peroxynitrous acid (ONOOH)^12^. Stimulated neutrophils, monocytes and some tissue macrophages release myeloperoxidase that utilises H_2_O_2_ to generate powerful two-electron oxidants [e.g. hypochlorous acid (HOCl), hypobromous acid (HOBr), hypothiocyanous acid (HOSCN)] and radicals^13^,^14^. These processes are critical to efficient pathogen killing, but result in significant collateral damage to host tissues^15^. Oxidant formation can also arise via electron-leakage from mitochondria, from enzymes such as xanthine oxidase and lipoxygenases, and by uncoupling of enzymes such as endothelial and inducible nitric oxide synthases (NOS) (reviewed)^8^. Exogenous factors can also generate oxidants, including radiation and sunlight exposure (via singlet oxygen, ^1^O_2_, formation), metal ions, and environmental pollutants (e.g., chemicals, drugs, solvents, particulates, cigarette smoke). Consequently, chronic inflammation and increased oxidant levels have been linked with multiple human pathologies^8^.

Radical-mediated damage to biological targets has been studied extensively, but less is known about the reactions of HOCl, HOBr, HOSCN, ONOOH and ^1^O_2_^12^,^14^,^15^,^16^. Proteins are major targets for these oxidants as a result of their abundance, and high rate constants for reaction, with sulfur-containing amino acids being particularly prone to modification due to the presence of the reactive sulfur center (reviewed)^15^,^17^,^18^. Oxidation of cysteine (Cys) and methionine (Met) residues is relatively well understood (reviewed)^19^,^20^, but modification of disulfides (e.g. cystine), and the factors that control this, are less well characterized, despite the critical importance of such bonds in maintaining protein structures. Disulfide bond modification is postulated to be a key factor in determining the shelf life and activity of protein- and peptide-based medicines including antibodies and vaccines^21^,^22^, in food processing and spoilage^23^, in amyloid and aggregate formation^24^,^25^ and in some human diseases^26^,^27^.

In the present study we show that the apparent second-order rate constants (*k*_*2*_) for oxidation of disulfide bonds (both low molecular mass and also in proteins) vary by up to 10^4^-fold, and with multiple oxidants, with the variations arising from interactions between the reaction center and nearby heteroatoms. Theoretical computations have been used to help rationalize the experimental observations.

## Results

### Apparent second-order rate constants for reaction of HOCl with linear (acyclic) disulfides

Rate constants (*k*_*2*_) for reactions of HOCl with acyclic disulfides were determined under pseudo first-order conditions, with the disulfide in excess, using direct stopped-flow with UV detection (230–320 nm). The resulting data were analyzed by either plotting *k*_*obs*_ values determined from a single exponential fit (Pro-Data Viewer) to single wavelength data (between 230–310 nm) against the disulfide concentration, or by global analysis over the complete spectral range to directly determine *k*_*2*_. Kinetic data were obtained at pH 7.4 and 10 °C, and for a limited number of the disulfide compounds also at 22 °C, with the lower temperature used to slow the reactions and enhance the accuracy of the data.

**Figure 1:**
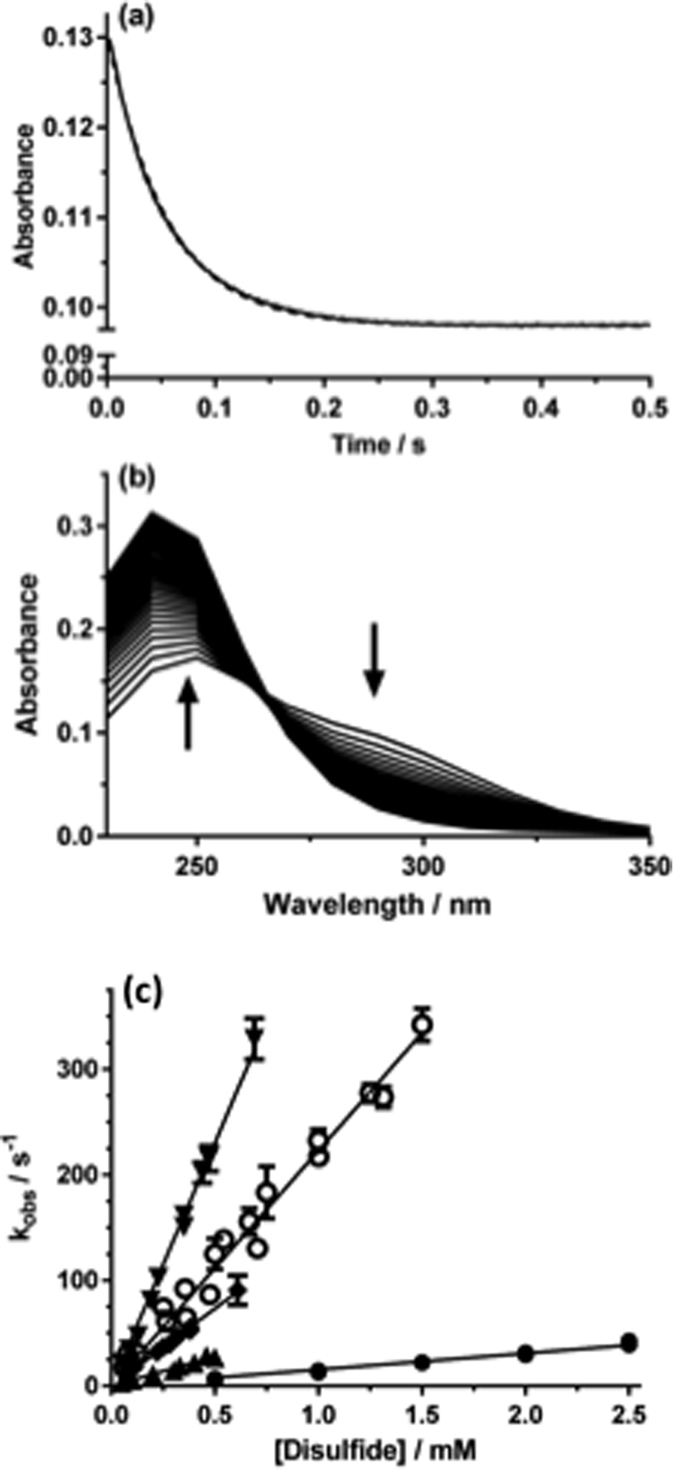
Kinetic data obtained for the reaction of disulfides with HOCl. (**a**) Decrease in absorbance at 270 nm (solid line) detected on reaction of bis-(2-hydroxyethyl) disulfide (2-OH-SS; 1 mM) with HOCl (0.5 mM) in phosphate buffer (0.1 M, pH 7.4) at 22 °C, with the corresponding exponential fit (dotted line). (**b**) Time dependent spectral changes for the reactions indicated in (**a**) over the spectral range 230–350 nm at 10 nm intervals over 0.5 s. (**c**) Pseudo-first order plots of *k*_*obs*_ versus substrate concentration for reaction of disulfides and HOCl in phosphate buffer (0.1 M, pH 7.4, 10 °C). Data for *trans*-4,5-dihydroxy-1,2-dithiane (4,5-(OH)_2_-dithiane, ▲), Gly-Cys (▼) and GSSG (◆) were determined with 50 μM HOCl, whilst those for cystamine (○) and 2-OH-SS (●) used 0.5 mM HOCl to improve signal intensity. Each data point is *k*_*obs*_ from an individual sample, with the error bars representing the standard deviation from n > 3 fresh aliquots.

**Figure 2:**
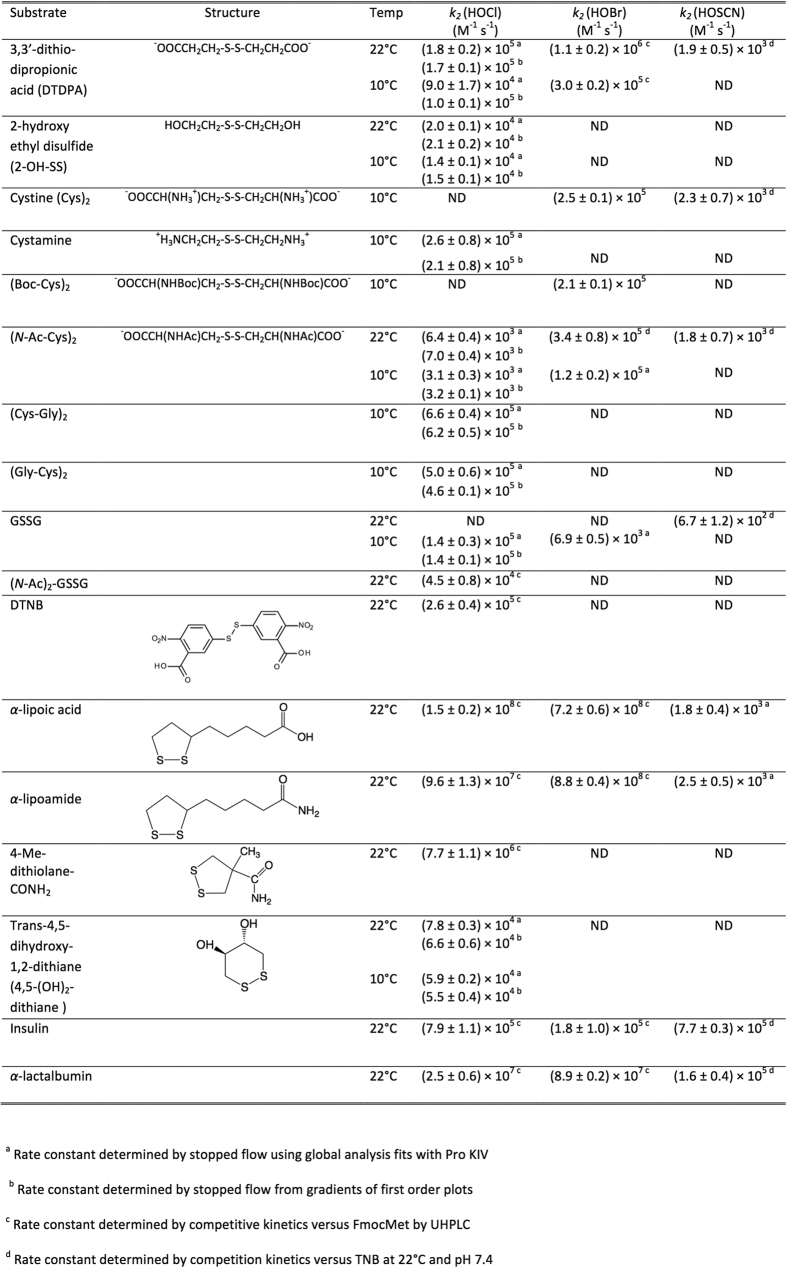
Apparent second order rate constants for reaction of oxidants with disulfide bonds.

*k*_*2*_ for reaction of HOCl with the acylic disulfide 3,3′-dithiodipropionic acid (DTDPA) was determined as (1.7 ± 0.1) × 10^5^ M^−1 ^s^−1^, consistent with previous results [(1.8 ± 0.2) × 10^5^ M^−1 ^s^−1^ at 22 °C and pH 7.4]^28^. At 10 °C, quenching plots gave *k*_*2*_ (1.04 ± 0.09) × 10^5 ^M^−1 ^s^−1^, and global analysis (9.0 ± 1.7) × 10^4 ^M^−1 ^s^−1^. Analogous experiments with other acyclic disulfides including bis-(2-hydroxyethyl) disulfide (2-OH-SS), cystine, cystamine, (*N*-Ac-Cys)_2_, (Cys-Gly)_2_, (Gly-Cys)_2_ and GSSG (Fig. 1) gave *k*_*2*_ values between 6 × 10^3 ^M^−1 ^s^−1^ [for (*N*-Ac-Cys)_2_] and 4.5 × 10^4 ^M^−1 ^s^−1^ (for (*N*-Ac)_2_-GSSG) at 22 °C. The values at 10 °C were lower as expected. All the resulting rate constants are presented in Fig. 2. *k*_*2*_ for reaction of HOCl with (*N*-Ac)_2_-GSSG (due to limited availability) and 5,5′-dithiobis-(2-nitrobenzoic acid) (DTNB; where the absorbance bands of HOCl and DTNB overlap) were determined using competition kinetics with Fmoc-Met (methionine tagged with the fluorescent Fmoc group on the *N*-terminus) as the competitive substrate, with the yields of the parent (Fmoc-Met) and its oxidation product, Fmoc-Met sulfoxide (Fmoc-MetSO) determined by ultra performance liquid chromatography (UPLC) analysis^28^.

### Apparent second-order rate constants for reaction of HOCl with cyclic disulfides

**Figure 3:**
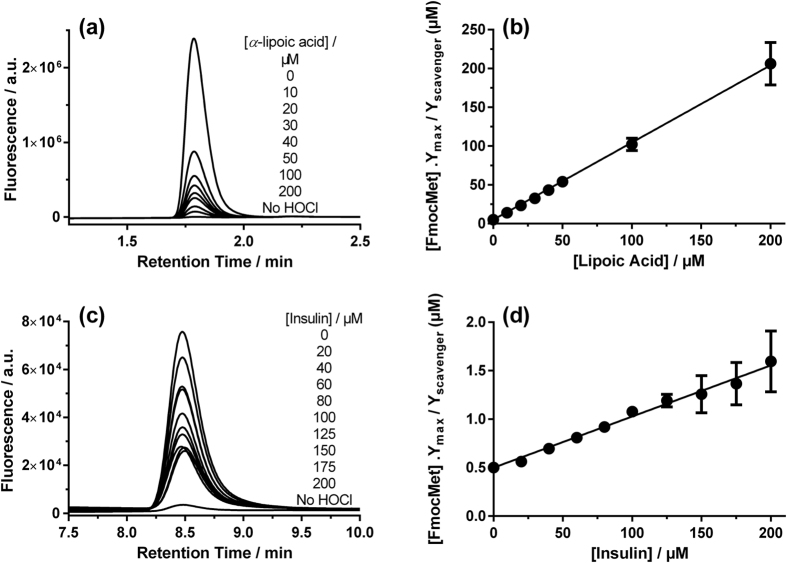
Competitive kinetic analysis of the reactions of (**a**,**b**) HOCl (2 μM) with α-lipoic acid and Fmoc-Met (5 μM) and (**c**,**d**) HOCl (0.2 μM) with insulin and Fmoc-Met (0.5 μM). Conversion of Fmoc-Met to Fmoc-Met sulfoxide (Fmoc-MetSO; peaks shown in **a**,**c**) was monitored by UPLC, with data from a single representative experiment shown. Plots (**b**,**d**) show data (mean ± SEM) from at least six replicates.

Rate constants for cyclic disulfides were determined using competition kinetics due to the rapidity of these reactions (e.g. Figs 2 and 3a,b). *k*_*2*_ for the 6-membered ring compound *trans*-4,5-dihydroxy-1,2-dithiolane was determined as (5.9 ± 0.2) × 10^4^ M^−1 ^s^−1^ (10 °C, pH 7.4), in line with the data for many of the acyclic disulfides (Fig. 2). In contrast, the 5-membered ring compounds α-lipoic acid, α-lipoamide, and 4-methyl-1,2-dithiolane-4-carboxamide (4-Me-dithiolane-CONH_2_) yielded *k*_*2*_ values of (1.5 ± 0.2) × 10^8^, (9.6 ± 1.3) × 10^7^ and (7.7 ± 1.1) × 10^6^ M^−1 ^s^−1^, respectively, with these being up to 20,000-fold higher than for the acyclic disulfides (Figs 2 and 3).

### Apparent second-order rate constants for reaction of HOCl with proteins

**Figure 4:**
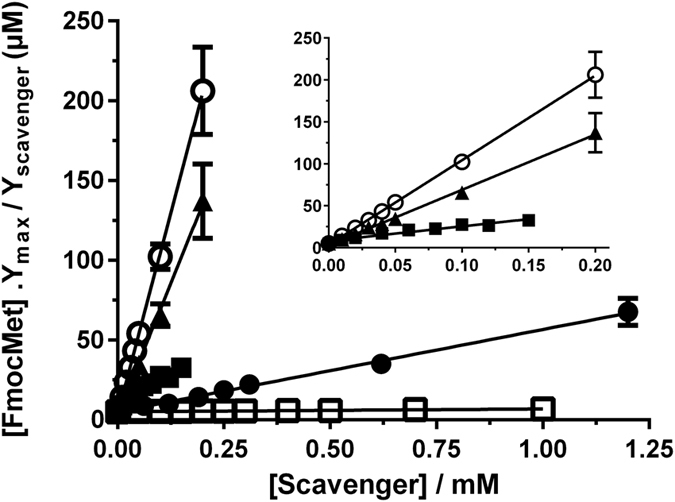
Plots derived from competitive kinetic analysis of the reactions of HOCl (2 μM) with α-lipoic acid (○), α-lipoamide (▲), lactalbumin (◼), 4-Me-dithiolane-CONH_2_ (⚫) or DTNB (□) in the presence of Fmoc-Met (5 μM). Data are mean ± SEM from six replicates (where error bars cannot be seen they are smaller than the symbol). Inset shows data on an expanded x-axis for α-lipoic acid (**○**), α-lipoamide (**▲**), lactalbumin (**◼**), showing linearity even at low scavenger concentrations.

Rate constants were determined for reaction of HOCl with insulin and α-lactalbumin, which contain multiple disulfide bonds but do not contain any free Cys residues that would be a competitive target for this oxidant. The values of *k*_*2*_ determined (at 22 °C, pH 7.4) using competition kinetics were (7.9 ± 1.1) × 10^5^ M^−1 ^s^−1^ for insulin (Figs 2 and 3c,d) and (2.5 ± 0.6) × 10^7^ M^−1 ^s^−1^ for α-lactalbumin (Figs 2 and 4).

### Apparent second-order rate constants for reaction of HOBr and HOSCN with disulfides

In the light of the variation in *k*_*2*_ values for HOCl, similar experiments were carried out with HOBr and HOSCN and a limited number of disulfides, using either direct stopped-flow experiments or competition kinetics. As with HOCl, a marked enhancement in *k*_*2*_ was observed with the 5-membered cyclic disulfides, α-lipoic acid and α-lipoamide with HOBr (Fig. 2), whereas with the less reactive oxidant HOSCN, no significant enhancement was detected. High *k*_*2*_ values were also determined for reaction of HOBr with α-lactalbumin, and for HOSCN with both proteins (Fig. 2).

### Computational modeling of reactions

The variation in *k*_*2*_ for the acylic disulfides (~10^2^), and between the acyclic species/6-membered ring structures and the 5-membered rings (~10^4^), prompted investigation of the possible reasons for this variation. Calculation of the thermodynamics of reaction of a range of disulfide structures with HOCl, assuming the mechanism illustrated in equation 1, was carried out using the MO6-2X method and the SMD continuum model for solvent water.


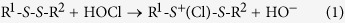


**Figure 5:**
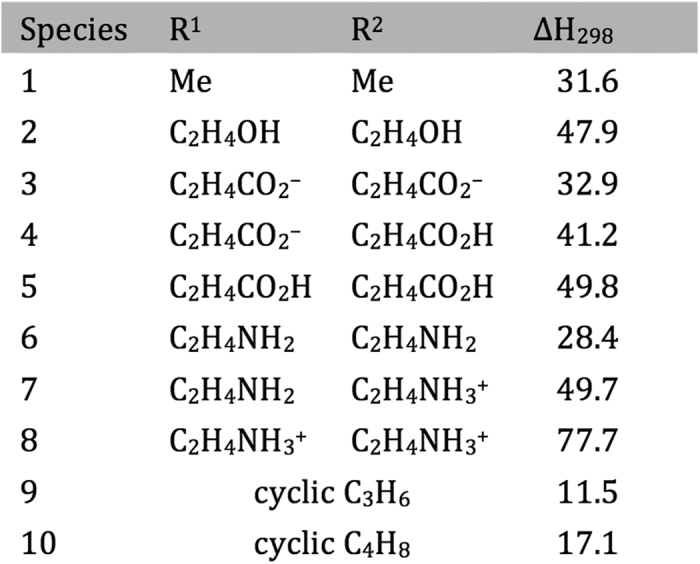
Calculated reaction enthalpies in kJ mol^−1^ for the reaction: R^1^-SS-R^2^(aq) + HOCl(aq) → R^1^-S^+^(Cl)S-R^2^ (aq) + HO^−^(aq).

The resulting calculated reaction endothermicities at 298 K (Fig. 5) show considerable variation with both the nature and ionization state of the included functional groups (carboxylic acid/anion, neutral and protonated amine, and hydroxyl, chosen to model some of the compounds included in Fig. 2), and whether R^1^ and R^2^ were linked in a 5- or 6-membered ring. The 5-membered ring structure had the lowest calculated endothermicity (11.5 kJ mol^−1^), followed by the 6-membered ring (17.1 kJ mol^−1^). For the acyclic species, the neutral diamine (neutral cystamine) had the lowest value (28.4 kJ mol^−1^), with this increasing markedly with both single (49.7 kJ mol^−1^) and double (77.7 kJ mol^−1^) protonation, consistent with expectations based on an increasingly unfavorable interaction with the positively-charged sulfur center. Variation in the calculated values also occurs with the dicarboxylic acid species, 3,3′-dithiodipropionic acid (DTDPA), with the lowest values arising from the di-anion, with these increasing on mono- and di-protonation to the neutral carboxylic acid. The variation in this case is much less marked than with the amine substituents (~17 kJ mol^−1^ for the anion/acid, compared with ~50 kJ mol^−1^ for the amine series). The endothermicity calculated for the dicarboxylate is considerably lower than for the di-hydroxyl species (2-OH-SS) (32.9 versus 47.9 kJ mol^−1^), but comparable to the calculated value for dimethyldisulfide (31.6 kJ mol^−1^), which is neutral and does not include the strongly electron-withdrawing OH groups. These data are also consistent with interaction with the positively-charged sulfur center being increasingly favorable in going from neutral to monoanionic to dianionic substituents.

**Figure 6:**
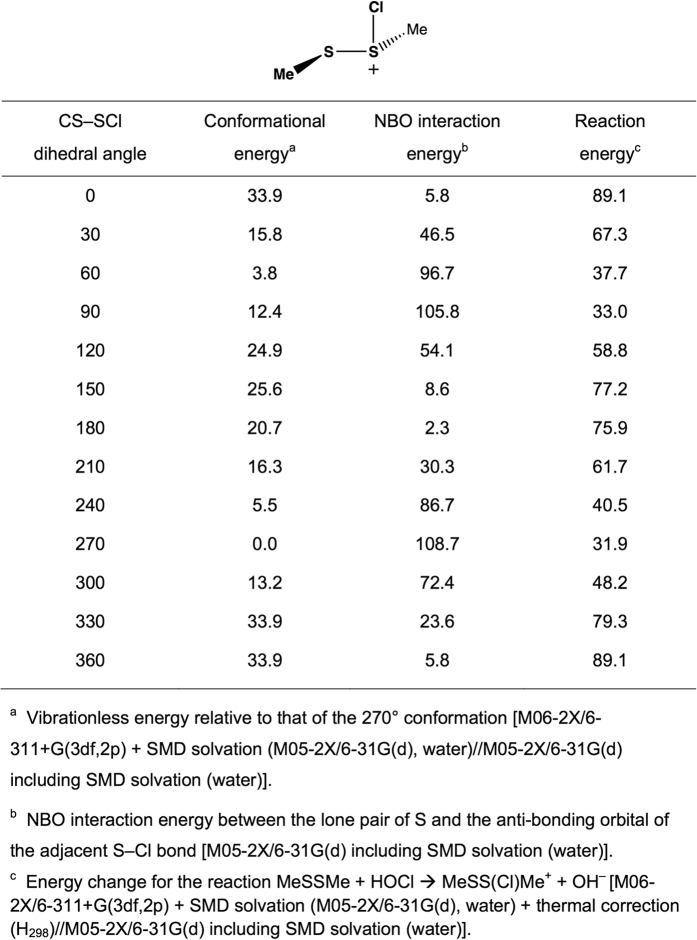
Conformational energies, ^a^NBO interaction energies, ^b^and reaction energies^c^ (kJ mol^−1^) calculated as a function of the CS–SCl dihedral angle in MeSS(Cl)Me^+^.

**Figure 7:**
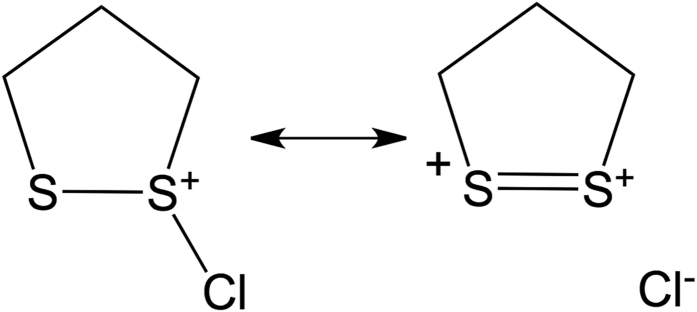
Stabilization by electron donation from the sulfur lone pair into the antibonding S–Cl orbital.

The role of ring conformation in modulating the reaction kinetics was investigated by initially examining the CH_3_S–^+^S(Cl)CH_3_ system as a model for the chlorine adduct. In particular, quantum chemistry computations at the M05-2X/6-31G(d) level in conjunction with the solvation model based on density (SMD) continuum solvation model, were used to calculate geometries as a function of the CS–SCl dihedral angle. The variation in vibrationless energy as a function of the CS–SCl dihedral angle was then obtained through single-point calculations at these geometries at the M06-2X/6-311 + G(3df,2p) + SMD solvation (M05-2X/6-31G(d), water) level. The data presented in Fig. 6 indicate that the preferred conformations correspond to CS–SCl dihedral angles of between 60–90° and approximately 270°. The fully optimized structures have dihedral angles of 69° and 273°, respectively, with the latter lying lower in energy by 4.6 kJ mol^−1^. In such conformations, the p-type lone pair on sulfur is approximately parallel to the S–Cl bond. Such conformations facilitate stabilizing electron donation from the sulfur lone pair into the antibonding S–Cl orbital (Fig. 7), analogous to the interaction responsible for the anomeric effect in carbohydrate chemistry^29^,^30^,^31^.

**Figure 8:**
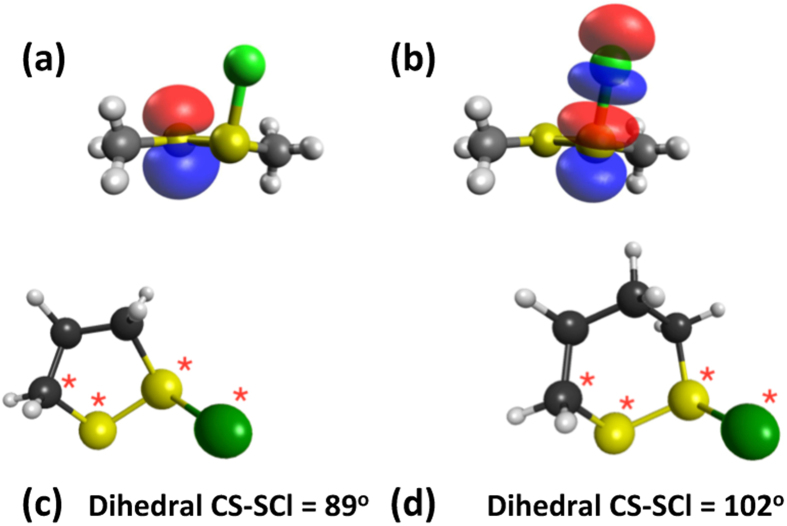
Schematic representation of the interaction between natural bond orbitals corresponding to (**a**) the sulfur lone pair and (**b**) the antibonding orbital of the adjacent S–Cl bond (M05-2X/6-31 G (d) with SMD continuum solvation), and optimized structures of the chlorine adducts for (**c**) the 5-membered ring and (**d**) the 6-membered ring (M05-2X/6-31 G (d) with SMD continuum solvation).

In order to quantify such interactions, the natural bond orbital (NBO) method^32^ has been used to determine interaction energies between a sulfur lone pair and the antibonding orbital of the adjacent S–Cl bond (Figs 6 and 8). It can be seen that the best NBO interaction energies correspond to CS–SCl dihedral angles of 90° and 270°, in line with the best optimized structures. More generally, the NBO interaction energies correlate well with the calculated conformational energies (Fig. 6). Figure 6 also contains the calculated reaction energies for equation 2, with these data indicating that there is a good correlation between the calculated reaction energies and the calculated NBO interaction energies.


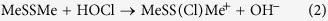


These data provide important insights into the behavior of the 5- and 6-membered ring systems. We start by noting that the calculated CS–SCl dihedral angles are 89° in the 5-membered ring adduct and 102° in the 6-membered ring adduct (Fig. 8c,d). The computed NBO interaction energies are 100.5 and 83.7 kJ mol^−1^, respectively. These results are consistent with expectations based on the results for the model system: the better alignment of the orbitals in the 5-membered ring adduct (dihedral angle very close to the optimum 90°) results in a more favorable interaction energy than in the 6-membered ring adduct (dihedral angle of 102°).

The reactivity of the 5- and 6-membered ring systems with HOCl can therefore be rationalized in terms of the preferred conformations. Specifically, the structural constraints in the cyclic systems lead to more effective electronic interaction between the sulfur lone-pair and the antibonding orbital of the adjacent S–Cl bond in the chlorine adduct of the 5-membered ring than in the 6-membered ring.

## Discussion

Sulfur-centered amino acids, both free and in peptides/proteins, are major targets for one-electron (radical, e.g., HO^⋅^, RO^⋅^, ROO^⋅^) and two-electron (e.g., H_2_O_2_, ONOOH, HOCl, HOBr, HOSCN, ^1^O_2_, O_3_) oxidants^20^,^33^. Multiple studies have shown that Cys and Met residues (both free, and in proteins) are rapidly oxidized, and are major targets^20^,^33^,^34^. In contrast relatively little is known about the oxidation of cystine (free or in proteins), with most studies focused on -S-S- bond reduction. The limited data available indicate that thiosulfinates (R-S-S(=O)-R’; disulfide S-oxides) are the major initial oxidation products^35^,^36^,^37^,^38^, but that these react further, resulting in -S-S- bond cleavage and sulfonic acid (RSO_3_H) formation. Thiosulfinates have been detected on some low-molecular-mass disulfides and proteins^35^,^36^,^37^,^38^. These are thought to arise via initial adduct (-S-S^+^(X)-) formation and subsequent rapid hydrolysis (equation 3, X = Cl, Br, SCN), with the latter reaction being rapid (due to the high concentration of water in most biological systems) and irreversible.


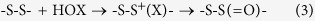


The biological significance of disulfide oxidation has been previously unclear, due to the paucity of kinetic data for these reactions. We have reported previously that acyclic disulfides react with HOCl and HOBr with moderate *k*_*2*_ values (1–3 × 10^5^ M^−1 ^s^−1^;^39^,^40^), but the effects of structure and electronic factors had not been elucidated, though it has been noted that ONOOH reacts with α-lipoic acid more rapidly than with GSSG and cysteine^41^,^42^. Considerable variation has, however, been reported for disulfide reactions with ^1^O_2_^43^. The reactivity of Cys with oxidants is known to be pH and environment sensitive, with this arising partly from the increased reactivity of the thiolate (RS^−^) over the neutral form^33^, but also from electronic and structural factors [cf. *k*_*2*_ for reaction of H_2_O_2_ with the active site Cys of peroxiredoxins of ~10^7^ M^−1^ s^−1^, compared with ~3 M^−1^ s^−1^ for the Cys-34 residue of BSA]^33^,^44^.

The current study shows that disulfides also show large variations in *k*_*2*_, but for alternative reasons. For acyclic species, this variation is ~2 orders of magnitude, with this increasing to >4 orders of magnitude when the acyclic species are compared with the 5-membered ring disulfides. Of particular note is the increased reactivity associated with stabilization by appropriately positioned heteroatoms, and the much higher values for 5-membered ring species compared with 6-membered rings and acyclic species. For the acyclic species, this increased reactivity arises from interactions of suitably positioned electron-donor groups with the incipient positive charge developed at the sulfur reaction center, resulting in stabilization of this species (cf. Fig. 7). Enhanced reactivity is also seen with HOBr, and to a lesser extent HOSCN with proteins. The smaller effects seen with HOSCN at this pH (7.4), may be associated with differences between the attacking species, with this being the *neutral* species for HOCl and HOBr (pK_a_ 7.6 and 8.7, respectively ref. 14), and ^**−**^OSCN for HOSCN (pK_a_ 4.85)^45^. However, even with ^**−**^OSCN, a significant enhancement is apparent, as *k*_*2*_ values for other disulfides are too low to be measured accurately (i.e., ≪10^3^ M^−1^ s^−1^)^46^. These data, together with the computed reaction endothermicities, indicate significant and complex pH dependencies for these reactions arising from the protonation/deprotonation equilibria of both the oxidant and the target disulfides (Fig. 5). Thus the local environment of a disulfide markedly affects its reactivity.

**Figure 9:**
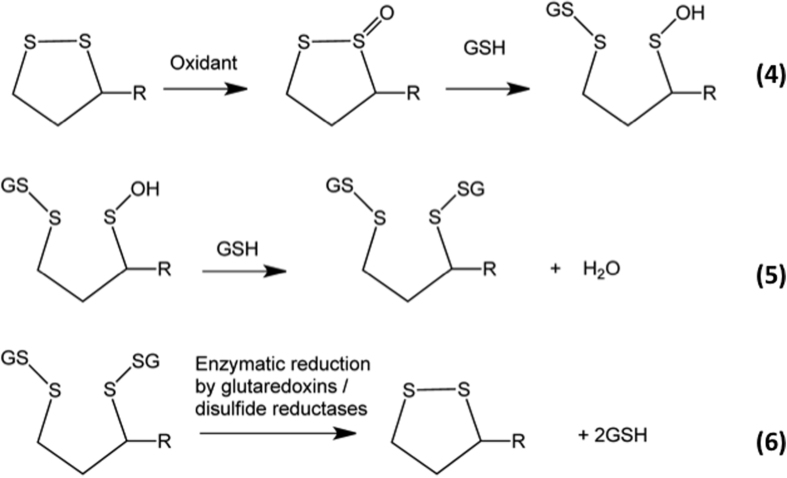
Equations 4–6.

Disulfide bond *conformation* also has a dramatic effect and drives the large increases in *k*_*2*_ between 5- versus 6-membered rings; these differences are consistent with the calculated endothermicities. This enhanced reactivity is rationalized in terms of a highly-favorable conformation of the reactant that maximizes overlap of the p-orbitals of the sulfur atom with those of the appropriate ^+^S-X antibonding orbital at the adjacent sulfur reaction center allowing considerable hyperconjugative stabilization. The corresponding orbitals in the 6-membered ring do not allow this stabilization as effectively. These data are consistent with the proposal^47^,^48^ that α-lipoic acid and α-lipoamide are significant targets for oxidants, and protective agents against damage to proteins with which they are associated^49^. Whether the resulting thiosulfinates can be repaired *directly* is not established, though these can undergo further reaction with GSH to give mixed disulfides and sulfenic acids, which can be repaired^50^ (equations 4ȓ6, Fig. 9). Disulfide oxidation to a thiosulfinate, and subsequent reaction with GSH, may therefore act as a *catalytic* oxidant-scavenging pathway. This pathway also provides a route to protein glutathionylation, a process strongly implicated in cellular signaling events^51^,^52^, for proteins that lack free Cys residues.

Comparison of the kinetic data obtained for the low-molecular-mass disulfides and proteins, indicates that protein disulfides can also show increased reactivity. Both the proteins studied contain multiple disulfides. For insulin, there are two inter-chain disulfides (A7-B7 and A20-B19) between the A and B chains, and one intra-chain linkage (A6-A11). Each is important for the receptor binding activity of this protein, with A20-B19 the most important, and A6-A11 bond the least^53^. The value of *k*_*2*_ determined for reaction of HOCl with insulin is significantly larger than that determined for (*N*-Ac-Cys)_2_ and 2-OH-SS, slightly faster than others (such as DTDPA and GSSG), and comparable with that for cystamine. These data suggest that protein-dependent factors enhance the reactivity of one (or more) of the disulfides in the protein, though the current data do not allow identification of which. A similar increased reactivity was seen with HOSCN (*k*_*2*_ 7.7 × 10^5^ M^−1 ^s^−1^ for insulin, compared with ~10^3^ M^−1 ^s^−1^ for the model compounds), but not with HOBr.

Bovine α-lactalbumin has four disulfide bonds that are important in protein aggregation and thiol-disulfide exchange reactions^54^. The *k*_*2*_ values for reaction of this protein with HOCl, HOBr and HOSCN are markedly higher than for the acyclic disulfides. Although α-lactalbumin contains Met residues, which may contribute to the increased reactivity with HOCl and HOBr, this cannot account for the HOSCN data, as this oxidant does not react significantly with Met^46^. The increases in *k*_*2*_ are consistent with one (or more) of the α-lactalbumin disulfides being present in a conformation that favors orbital overlap and stabilization of the incipient charged sulfur center, in a manner similar to the 5-membered ring compounds.

The conclusion that favorable conformations result in rapid reaction is consistent with data indicating that some protein disulfides exist in strained conformations^5^,^6^. These structures are particularly abundant in proteins with catalytic disulfides (e.g. thioredoxins) or those that have allosteric actions^6^,^55^. These previous studies have focused on disulfide reduction, rather than oxidation as examined here, but it is clear that there are factors that enhance both reactions. These may differ and warrant further study. Irrespective of this, it is clear that the reactivity of protein disulfides is dramatically affected by local structure and conformation. As disulfides are critical to maintaining protein structure, the current data indicate that some proteins, and sites within proteins, are particularly prone to oxidative modification and loss of function. Further studies are clearly warranted to extend these observations to a wider range of proteins, and also to proteins present in both folded and unfolded states.

Disulfide oxidation may be of particular relevance for *extracellular* proteins, as these typically have few Cys, large numbers of cystines, and modest numbers of Met residues. This is exemplified by human serum albumin, which has a single Cys, 6 Met and 17 disulfides (cf. UniProt entry P02768), and many extracellular matrix proteins (e.g., laminins, fibrillins and fibulins). Many extracellular matrix protein disulfides have critical functional roles, particularly with regard to the formation of receptor structures and epidermal growth factor (EGF) domains. Laminins are major components of most basement membranes, and are critical determinants of tissue architecture and cell binding, and contain up to 12% cystine residues. Fibrillins have even higher abundances, with fibrillin-1 containing 47 EGF–like domains each containing three conserved cystine bridges, with mutations in these cystine bridges associated with Marfan syndrome, the most common disorder of connective tissues^56^,^57^. Fibulins are also exceptionally cystine-rich, with this protein containing both large numbers of similar EGF domains, and an Arg-Gly-Asp (RGD) sequence critical for cell adhesion through interactions with integrins. As such, it has been strongly associated with cardiovascular disease^58^ and protection against cancer cell metastasis^59^.

## Conclusions

Overall, the data presented here suggest that oxidant-mediated damage to disulfides in proteins can be particularly rapid and may play a critical role in tissue dysfunction. This is likely to be particularly true for extracellular proteins, or domains, which are cystine-rich and can be exposed to high levels of oxidants, as a result of the low levels of extracellular defense and repair systems, and the long half-life and slow turnover of extracellular matrix proteins and proteoglycans.

## Materials and Methods

### Reagents

(±) α-Lipoic acid, 3,3′-dithiodipropionic acid (DTDPA), bis-(2-hydroxyethyl) disulfide (2-OH-SS), L-glutathione disulfide (GSSG), cystamine dihydrochloride, *trans*-4,5-dihydroxy-1,2-dithiane (4,5-(OH)_2_-dithiane), 5,5′-dithiobis-(2-nitrobenzoic acid) (DTNB), α-lactalbumin and recombinant human insulin (from *Saccharomyces cerevisiae*) were obtained from Sigma-Aldrich (Castle Hill, NSW, Australia). Fmoc-Met, Fmoc-MetSO, and the disulfides (*N*-Ac-Cys-OH)_2_, (H-Cys-Gly-OH)_2_, (H-Gly-Cys-OH)_2_, (Boc-Cys)_2_ were from Bachem (Bubendorf, Switzerland). (±) α-Lipoamide was obtained from Santa Cruz Biotechnology (Santa Cruz, CA, USA). di-*N*-acetyl-glutathione disulfide [(*N*-Ac)_2_-GSSG] was from Auspep (Tullamarine, Vic, Australia). 4-Methyl-1,2-dithiolane-4-carboxamide (4-Me-dithiolane-CONH_2_) was from Ambinter (GreenPharma, Orleans, France). Samples were prepared in 0.1 M phosphate buffers (pH 7.4) using Nanopure water (four-stage Milli-Q system; Millipore, NorthRyde, Australia). Buffers were treated with washed Chelex resin (Bio-Rad, Gladesville, NSW, Australia) to remove contaminating transition metal ions.

### Stopped-flow spectrophotometry

Apparent second-order rate constants (*k*_*2*_) for reactions of oxidants with disulfides were obtained either by directly monitoring changes in absorbance using a stopped-flow system, or via competition kinetic methods using UPLC as reported previously^28^,^60^ and described in the Supplementary Information.

### Competition kinetic measurements for reaction of HOCl and HOBr with disulfides using UPLC

Competition kinetic data were obtained for HOCl and HOBr using the conversion of Fmoc-Met to Fmoc-Met sulfoxide (Fmoc-MetSO) as the reference reaction as described previously^28^,^60^ and in the Supplementary Information.

*k*_*2*_ for reaction of Fmoc-Met with HOBr was determined using DTDPA as the reference reaction [*k*_*2*_ (1.1 ± 0.2) × 10^6^ M^−1 ^s^−1^ at 22 °C]^40^, yielding a value at 22 °C and pH 7.4 of (6.5 ± 0.1) × 10^8^ M^−1 ^s^−1^. This value was used to determine the values for the disulfides as described above for HOCl. As with HOCl, the primary oxidation products of HOBr and disulfides do not oxidize Fmoc-Met, and did not change the initial yield of Fmoc-MetSO. Details of the separation and detection methods are given in the Supplementary data.

### Competition kinetic experiments for reactions of HOSCN

Rate constants for the reactions with HOSCN were determined using competition kinetics with oxidation of 5-thio-2-nitrobenzoic acid (TNB) to 5,5′-dithiobis-(2-nitrobenzoic acid) (DTNB) as the reference reaction^46^. HOSCN (2.5 μM) was kept as the limiting reagent with a minimum 4-fold excess of disulfide and a constant concentration of TNB (10 μM). Concentrations of TNB were determined at 412 nm, using ε 14150 M^−1 ^cm^−1^ 46.

#### Molecular modelling calculations

Standard density functional theory calculations were carried out using Gaussian 09^61^. Geometry and vibrational frequency calculations were carried out at the M05-2X/6-31G(d) level^62^ in the presence of a solvent (water) continuum computed with the SMD model^63^. Zero-point vibrational energies and enthalpic temperature corrections were obtained from scaled M05-2X/6-31G(d) vibrational frequencies using literature scale factors^64^. Single-point energy calculations were carried out with M06-2X/6-311 + G(3df,2p) + SMD solvation + thermal corrections^65^. Relative energies are quoted in kJ mol^−1^ and they represent 298 K condensed-phase enthalpies. Analysis of interactions was carried out using the natural bond orbital procedure^32^.

### Errors and statistics

Stopped-flow data (reported with 95% confidence limits) were obtained from at least two independent experiments each using typically 5 different substrate concentrations, with 10 kinetic traces averaged at each concentration. For competition kinetic data (reported with 95% confidence limits) the experiments were repeated at least 3 times, with duplicate samples for each experiment. Prism 5.0 (v3.0; GraphPad, Inc) or Origin Pro 8.0 (Origin Lab, Northampton, MA, USA) were used for statistical analyses of the linear fits. Standard error propagation methods were used to combine the experimental uncertainties in *k*_*2*_ and the slopes of the competition plots to provide the final confidence intervals for the reported *k*_*2*_ values.

## Additional Information

**How to cite this article**: Karimi, M. *et al*. Reactivity of disulfide bonds is markedly affected by structure and environment: implications for protein modification and stability. *Sci. Rep.* **6**, 38572; doi: 10.1038/srep38572 (2016).

**Publisher's note:** Springer Nature remains neutral with regard to jurisdictional claims in published maps and institutional affiliations.

## Supplementary Material

Supplementary Data
