# Supplementary material for: Reactivity of disulfide bonds is markedly affected by structure and environment: implications for protein modification and stability
Source: Sci Rep. 2016 Dec 12;6:38572. doi: 10.1038/srep38572 (PMC5150571; doi:10.1038/srep38572)
Supplement: Supplementary Data [file srep38572-s1.pdf]

Supplementary data

Reactivity of disulfide bonds is markedly affected by structure and environment: implications for protein modification and stability

Maryam Karimi [1, 2, a], Marta T. Ignasiak [3, a], Bun Chan [4], Anna K. Croft [5], Leo Radom [4], Carl H. Schiesser [6], David I. Pattison [1,2] and Michael J. Davies [1,2,3] *

[1] The Heart Research Institute, 7 Eliza St, Newtown, NSW, 2042, Australia;

[2] Faculty of Medicine, University of Sydney, NSW, 2006, Australia;

[3] Department of Biomedical Science, Panum Institute, University of Copenhagen, Blegdamsvej 3, Copenhagen 2200, Denmark

[4] School of Chemistry, University of Sydney, Sydney, NSW 2006, Australia.

[5] Department of Chemical and Environmental Engineering, University of Nottingham, Nottingham NG7 2RD, Great Britain

[6] School of Chemistry, Bio21 Molecular Science and Biotechnology Institute, The University of Melbourne, Victoria 3010, Australia

[a] Contributed equally to this work

* To whom correspondence should be addressed at: Building 4.5, Panum Institute, Department of Biomedical Science, University of Copenhagen, Blegdamsvej 3, Copenhagen 2200, Denmark

Email: davies@sund.ku.dk

Materials and methods

Stopped-flow spectrophotometry

For the direct measurements, an SX20 stopped-flow system (Applied Photophysics, Leatherhead, UK) with Pro-Data SX (version 2.2.12) software was used, with this fitted with a 1 cm path length cell and either a single wavelength monochromator (band width, ± 1.2 nm; slit width, 0.5 mm) with photomultiplier detection, or a photodiode array detector for monitoring multiple wavelengths simultaneously. Spectral data from 230 to 340 nm were typically achieved in a point-by-point and time-dependent manner by acquiring kinetic traces at 10 nm intervals with the photomultiplier over this wavelength region. To minimize the occurrence of secondary reactions, low concentrations of oxidant were used for all kinetic measurements, with the substrate typically present in at least a five-fold molar excess to provide pseudo first-order reaction conditions. Reaction temperatures were maintained at either 22 or 10 °C by using a thermostatted circulating water bath (Lauda-Brinkmann). Kinetic data were analyzed by both pseudo-first-order and global analysis methods. For pseudo-first order analysis the exponential decays at the specified wavelength were fitted using Pro-Data Viewer (version 4.2.12; Applied Photophysics) whilst global analysis was carried out using Pro-Kineticist Mk IV (version 1.0.1.4; Applied Photophysics) with the fitting mechanism: oxidant + disulfide → oxidized disulfide.

Competition kinetic measurements for reaction of HOCl and HOBr with disulfides using UHPLC

Competition kinetic data were obtained for HOCl and HOBr using the conversion of Fmoc-Met to Fmoc-Met sulfoxide (Fmoc-MetSO) as the reference reaction as described previously (1, 2). This method is based on the measurement of a decreased conversion of parent Fmoc-Met to its oxidation product, which is formed in a stoichiometric manner, in the presence of increasing concentrations of a competing target for the oxidant. As the rate constant for oxidation of Fmoc-Met to Fmoc-MetSO by HOCl is well established (1, 2), and the concentrations of these two species can be readily determined by UHPLC with fluorescence detection of the Fmoc tag, the rate constant for reaction of the oxidant with the competitive target can be determined by standard competition analysis. The approach has been widely used and extensively validated (1, 2).

Separation of Fmoc-Met and Fmoc-MetSO was achieved using a Shimadzu Nexera UHPLC system with a Shimpack XR-ODS (Shimadzu, 100 x 4.6 mm, 2.2 μm) column maintained at 40 °C. The column was eluted at a rate of 1.2 mL min^{-1} for 12 min with gradient elution with a gradient of buffer A (50 mM sodium acetate, pH 5.3, in 20% methanol with 2.5% tetrahydrofuran), and buffer B (50 mM sodium acetate, pH 5.3, in 80% methanol with 2.5% tetrahydrofuran) as detailed previously (1, 2). Prior to injection (5 μl) samples containing low molecular mass disulfides were centrifuged to remove particulate matter (0.2 μm Pall Nano-sep MF filters; 2 min, 9300 g). For experiments using lactalbumin an additional filtration step (>3 kD Nanosep MF molecular weight cutoff filter; 10 min,16,000 g; Pall Corporation, Cheltenham, Vic, Australia) was added to the sample preparation to prevent fouling of the analytical UHPLC column with lactalbumin. The fluorescence of the Fmoc groups (λ_{ex} 265 nm; λ_{em} 310 nm) of Fmoc-Met and Fmoc-MetSO was used to monitor (Shimadzu RF-20AXS detector) the separation of Fmoc-MetSO (~ 1.8 min) and

Fmoc-Met (~ 3.5 min). Peak areas were determined using Lab Solutions 5.32 SP1 software (Shimadzu) and compared to authentic standards.

For the studies using recombinant human insulin, the method was modified as the 3 kD cutoff filters were not effective in removing excess insulin after the reaction due to the low molecular mass of this protein (*ca.* 5.8 kD). Thus the concentrations of Fmoc-Met and HOCl were decreased 10-fold (to 0.5 μM and 0.2 μM respectively), to allow the use of insulin concentrations (0 - 200 μM) that could be injected directly onto the analytical column without prior removal of the insulin, as insulin was found to bind to the filters leading to inconsistent Fmoc-Met and Fmoc-MetSO recovery. The samples were then incubated at 22 °C for 15 min before directly injecting onto the UHPLC system. Separation was achieved using a reverse-phase Zorbax C18 column (Agilent, 3.0 × 250 mm, 5 μm) at 30 °C with a flow rate of 0.4 mL min^{-1}. Samples were eluted with a gradient of buffer A (0.2% v/v trifluoroacetic acid in water) and buffer B (0.1% v/v trifluoroacetic acid in CH_3CN). The gradient profile started with 60% buffer A and 40% buffer B for 10 min, then increasing to 50% buffer B at 35 min, with a further increase to 100% B over the next 5 min and subsequent wash with 100% B for 8 min, before returning to 60% buffer A : 40% buffer B over 2 min and re-equilibration for 10 min, resulting in a total run time of 60 min. Under these separation conditions, fluorescence detection (λ_{ex} 265 nm; λ_{em} 310 nm; Shimadzu RF-20AXS detector) allowed Fmoc-MetSO (~ 8.5 min) and Fmoc-Met (~ 26 min), as well as insulin (~ 4 min), to be detected.

REFERENCES

1. Skaff O, *et al.* (2012) Selenium-containing amino acids are major targets for myeloperoxidase-derived hypothiocyanous acid: determination of absolute rate constants and implications for biological damage. *Biochem. J.* 441:305-316.
2. Storkey C, Davies MJ, & Pattison DI (2014) Reevaluation of the rate constants for the reaction of hypochlorous acid (HOCl) with cysteine, methionine, and peptide derivatives using a new competition kinetic approach. *Free radical biology & medicine* 73:60-66.
